# Modelling water use efficiency in a dynamic environment: An example using *Arabidopsis thaliana*^☆^

**DOI:** 10.1016/j.plantsci.2016.06.016

**Published:** 2016-10

**Authors:** S. Vialet-Chabrand, J.S.A. Matthews, O. Brendel, M.R. Blatt, Y. Wang, A. Hills, H. Griffiths, S. Rogers, T. Lawson

**Affiliations:** a School of Biological Sciences, University of Essex, Colchester, CO4 3SQ, UK; b EEF, INRA, Université de Lorraine, F-54280 Champenoux, France; c Laboratory of Plant Physiology and Biophysics, University of Glasgow, Bower Building, Glasgow G12 8QQ, UK; d Plant Sciences, University of Cambridge, Downing Street, Cambridge CB2 3EA, UK; e Computing Science, University of Glasgow, Alwyn Williams Building, Glasgow G12 8QQ, UK

**Keywords:** Stomatal conductance, Photosynthesis, Dynamics, Diurnal, Intrinsic water use efficiency

## Abstract

Intrinsic water use efficiency (*W_i_*), the ratio of net CO_2_ assimilation (*A*) over stomatal conductance to water vapour (*g_s_*), is a complex trait used to assess plant performance. Improving *W_i_* could lead in theory to higher productivity or reduced water usage by the plant, but the physiological traits for improvement and their combined effects on *W_i_* have not been clearly identified. Under fluctuating light intensity, the temporal response of *g_s_* is an order of magnitude slower than *A*, which results in rapid variations in *W_i_*. Compared to traditional approaches, our new model scales stoma behaviour at the leaf level to predict *g_s_* and *A* during a diurnal period, reproducing natural fluctuations of light intensity, in order to dissect *W_i_* into traits of interest. The results confirmed the importance of stomatal density and photosynthetic capacity on *W_i_* but also revealed the importance of incomplete stomatal closure under dark conditions as well as stomatal sensitivity to light intensity. The observed continuous decrease of *A* and *g_s_* over the diurnal period was successfully described by negative feedback of the accumulation of photosynthetic products. Investigation into the impact of leaf anatomy on temporal responses of *A*, *g_s_* and *W_i_* revealed that a high density of stomata produces the most rapid response of *g_s_* but may result in lower *W_i_*.

## Highlights

• A model scaling stoma behaviour at leaf level is proposed.
• The model is tested by reproducing natural variations of light intensity.
• Stomatal aperture and photosynthesis decrease as a function of sugar accumulation.
• Leaf anatomy influences the rapidity of stomatal conductance.
• The model dissects intrinsic water use efficiency into traits of interest.

## Nomenclature

*A*: Net CO_2_ assimilation
*A_G_*: Gross CO_2_ assimilation
*R_d_*: Mitochondrial respiration
*g_s_*: Stomatal conductance to water vapour
*g_m_*: Mesophyll conductance to CO_2_
*g_b_*: Boundary layer conductance to water vapour
*g_t_*: Total conductance to CO_2_
*W_i_*: Intrinsic water use efficiency
*C_a_*: Atmospheric CO_2_ concentration
*C_i_*: CO_2_ concentration in the intercellular airspaces
*C_c_*: CO_2_ concentration at the site of carboxylation
*a*: Stomatal pore area
*a_s_*: Steady state target of stomatal pore area
*a_min_*: Minimum stomatal pore area
*a_max_*: Maximum stomatal pore area
*α_L_*: Slope of the relationship
***θ***_***L***_: Curvature factor of the curve
*k_i_*: Time constant for an increase in *a*
*k*_*d*_: Time constant for an decrease in *a*
*L*: Percentage of efficiency
*SD*: Stomatal density
*D*: Diffusivity of water in air
*V*: Molar volume of air
*l*: Depth of stomatal pore
*P_a_*: The atmospheric pressure
*S_a_*: Factor representing the influence of the rate of accumulation of sugars
*S_e_*: Factor representing the influence of the rate of export of sugars
*Vc_max_*: Maximum Rubisco activity
*J_max_*: Maximum electron transport activity
α: Proportion of light absorbed by PSII

## 1 Introduction

In order to meet the projected demand for cereal production by 2050, crop yields must improve by 1.16–1.31% each year; however, current estimates are well below this required rate [1]. The primary determinant of crop yield is the cumulative rate of photosynthesis over the growing season and is determined by the ability of the plant to capture light and CO_2_, use this energy to convert the CO_2_ to biomass, and how much of this biomass ends in usable yield. Improving photosynthetic efficiency is recognised as an important but unexploited avenue to increase yield potential in crop plants [2]. Increasing photosynthetic efficiency is accompanied by a higher CO_2_ demand, which can be limited by the resistance of CO_2_ diffusion into the leaf. Any attempt to decrease this resistance greatly increases the water loss by transpiration from the leaf.

Photosynthetic productivity is linked to water consumed by the plant and often measured as water use efficiency (*WUE*). *WUE* can be defined at different scales of time and space and, at the leaf level, it is often assessed as the ratio of CO_2_ fixed per unit of H_2_O transpired (*E*). Intrinsic water use efficiency (*W_i_*) is defined when stomatal conductance to water vapour (*g_s_*) is used instead of *E*. The use of g_s_ to describe the stomatal control on the rate of *E* facilitates the comparisons between different leaves and environmental conditions. The photosynthetic capacity of the leaf determines the net CO_2_ assimilation (*A*) as a function of the variation in the micro-climate surrounding the leaf. Over the diurnal period, *A* is mainly determined by the irradiance absorbed by the leaf and the limitation of CO_2_ imposed by stomatal control. Under field conditions, environmental variables that affect both photosynthesis and stomatal behaviour are rarely constant. For example, light intensity (and spectral quality) alters in time scales of seconds to hours to which *A* and *g_s_* must respond. The temporal response of *A* and *g_s_* to a fluctuating environment are asynchronous, with *g_s_* response often an order of magnitude slower than *A*, which results in rapid variations of *W_i_*. Thus, it is important when describing the kinetic response of *W_i_* to use an approach that considers responses by *A* and *g_s_* simultaneously.

Intrinsic water use efficiency (*W_i_*) is dependent on the anatomy (e.g. stomatal size and density) and the physiology (e.g. behaviour) of stomata as well as the leaf biochemistry (e.g. activity of the Calvin cycle), all of which interact to determine the kinetics of CO_2_ and H_2_O gaseous exchange between the leaf and atmosphere. The dynamic nature of the interactions between the different components that determine *W_i_* are not fully understood and need to be addressed if we are to successfully improve both *A* and *W_i_* under dynamic field conditions.

It is possible to conceptualise the inherent complexity of gas exchange over a fluctuating light regime through modelling, which will improve our understanding of the *W_i_* response by simulating a number of gas exchange scenarios (e.g. changes in light intensity and humidity) that would normally be difficult to assess in a reasonable amount of time using experimental approaches. Current models focus on predicting g_s_ in steady state [3] and cannot be used to infer the impact of stomatal behaviour on *A* or *W_i_* under dynamic conditions. Although temporal responses of *g_s_* have previously been described using a dynamic model [4], [5], the relationship between stomatal response and leaf level gas exchange was not clearly described. We propose to use a model that will take into consideration the anatomy and physiology of stomata to more accurately represent the stomatal control of *W_i_*.

To scale stomatal responses to leaf level *g_s_*, the two most important stomatal characteristics are aperture and density [6], [7], [8]. A high stomatal density does not necessarily result in a higher g_s_ as stomata ultimately control their aperture depending on the guard cell responses to the external (e.g. light intensity) and internal (e.g. mesophyll demand for CO_2_) stimuli [9]. To link stomatal behaviour to leaf level gas exchange responses, we propose a ‘big stoma’ approach that consists of simulating the response of one stoma that is representative of the heterogeneous response of many stomata and scaling the response to the leaf level. This approach was incorporated in an enhanced version of the multi compartments model described by Noe and Giersch [10] to predict *A* and *W_i_*. Scaling up the dynamic of the stomatal response to the leaf level, with the improved model for CO_2_ diffusion inside the leaf, will help to dissect *W_i_* into traits of interest and predict potential gains in *W_i_*.

The objective of this study was to develop a new model combining our most recent knowledge of kinetics in stomatal behaviour and photosynthesis to describe the temporal response of *W_i_* over the course of a day with natural dynamic variations in irradiance. All the parameters of the model described here incorporate a trait of interest for *W_i_* and were adjusted using Bayesian inference. The model was validated using a dataset with a different irradiance pattern to assess the predictive power of the model. A sensitivity analysis was finally performed to show the interaction among the parameters and display the potential gain in *W_i_* in the case of one or two parameters changing. We used the output of the model to understand how temporal responses in *g_s_* impacts *A* and *W_i_*.

## 2 Material and methods

### 2.1 Dynamic modelling of photosynthesis and stomatal conductance

**Fig. 1:**
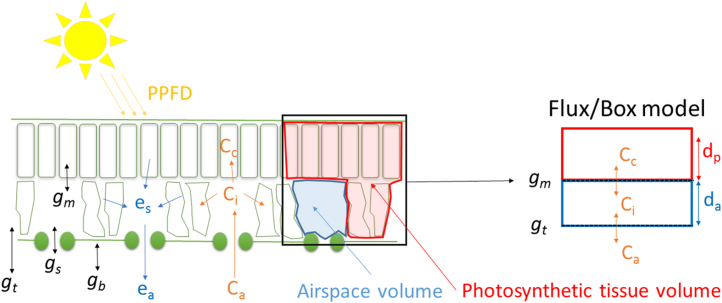
Schematic of the diffusion of gas exchange between the leaf and atmosphere compared to the conceptual version used in the model. The total conductance to water vapour (*g_t_*) controlling the gradient of H_2_O from leaf to atmosphere was composed by the stomatal conductance (*g_s_*) and the boundary layer conductance (*g_b_*). The gradient of CO_2_ from the atmosphere to the site of carboxylation was also dependent on mesophyll conductance (*g_m_*). The H_2_O concentrations inside and outside the leaf were represented by *e_s_* and *e_a_* respectively. The CO_2_ concentrations in three compartments were considered: in the atmosphere (C_a_), in the intercellular airspaces (C_i_) and at the sites of carboxylation (C_c_). The different compartments of the leaf were represented as a standardized volume defined by the thickness of the intercellular airspace (d_a_) and the photosynthetic tissues (d_p_).

The model essentially consists of four differential equations describing the diffusion of CO_2_ between different compartments represented by the atmosphere, the intercellular air spaces and the photosynthetic tissues (Fig. 1). The exchanges between these compartments are dependent on the stomatal aperture and the resistance of diffusion in the mesophyll cells. In addition, the model took into account the limitation of photosynthesis and stomatal aperture that appeared during a period of light.

(1) $$\{\begin{array}{c} \frac{da}{dt}=\frac{a_{s}-a}{k_{i}}ifa<a_{s}\\ \frac{da}{dt}=\frac{a_{s}-a}{k_{d}}ifa\geq a_{s} \end{array}$$

The first differential equation (Eq. (1)) described the temporal variations of the stomatal pore area (*a*) with *a_s_* the steady state target followed by *a* and two time constants, *k_i_* and *k*_*d*_, for an increase or a decrease of *a* respectively. Considering the spatial heterogeneity of the stomatal response, a top-down approach was used, signifying that the model simulated the response of one stoma representative of the sum of the individual stomatal responses and scaled it to leaf level instead of trying to integrate the response of each stoma.

The steady state target of *a* (*a_s_*) as a function of the light intensity (PPFD) was predicted using a non-rectangular hyperbola [4]:

(2) $$a_{s}=\left[a_{min}+\frac{\alpha_{L}PPFD+\left(a_{max}-a_{min}\right)-\sqrt{\alpha_{L}PPFD+{\left(a_{max}-a_{min}\right)}^{2}-4\theta_{L}\alpha_{L}PPFD\left(a_{max}-a_{min}\right)}}{2\theta_{L}}\right]\cdot L$$

with *a_min_* and *a_max_* the minimum and maximum stomatal pore area, α*_L_* the slope of the relationship, *θ*_*L*_ the curvature factor of the curve and *L* the percentage of efficiency (see below).

(3) $$\frac{dC_{i}}{dt}=\left[g_{t}\left(C_{a}-C_{i}\right)-g_{m}\left(C_{i}-C_{c}\right)\right]\frac{RT_{l}}{d_{a}P_{a}}$$

Eq. (3) described the variation of the CO_2_ concentration in the intercellular airspaces (*C_i_*) with *C_a_* the atmospheric CO_2_ concentration and *C_c_* the CO_2_ concentration at the sites of carboxylation.

The resistance of CO_2_ diffusion from the air to the leaf was represented by the total conductance to CO_2_ (*g_t_*):

(4) $$g_{t}=1/\left(1.6/g_{s}+1.37/g_{b}\right)$$

with *g_b_* the boundary layer conductance to water vapour estimated in the gas exchange chamber.

The stomatal conductance to water vapour (*g_s_*) was derived from *a* using the equation of Dow et al. [7]:

(5) $$g_{s}=\frac{SD\cdot D\cdot a}{V\left(l+\frac{\pi }{2}\sqrt{\frac{a}{\pi }}\right)}$$

with *SD* the stomatal density, *D* the diffusivity of water in air, *V* the molar volume of air and *l* the depth of the stomatal pore.

The resistance of CO_2_ diffusion in the mesophyll cells (*g_m_*) was calculated following the equations described by Von Caemmerer et al. [11] and the values given for *A*rabidopsis* thaliana*. As the temperature of the leaf was regulated during the experiment, *g_m_* was considered stable.

The last part of the equation $\frac{RT_{l}}{d_{a}P_{a}}$ was used to convert the fluxes (μmol m^−2^ s^−1^) between the two compartments (leaf and atmosphere) into concentrations of CO_2_ (μmol mol^−1^) contained in the volume represented by the intercellular airspaces (See the Appendix in Noe and Giersch [10] for more information), with *R* the gas constant, *T_l_* the leaf temperature, *P_a_* the atmospheric pressure, and *d_a_* the depth of the intercellular airspaces (*d*_*a*_ = leaf thickness (m) • airspaces (%)).

(6) $$\frac{dC_{c}}{dt}=\left[g_{m}\left(C_{i}-C_{c}\right)+R_{d}-A_{G}L\right]\frac{RT_{l}}{d_{p}P_{a}}$$

Eq. (6) described the variation of *C_c_* with *R_d_* the mitochondrial respiration during the day, *A_G_* the gross CO_2_ assimilation and *L* the efficiency coefficient of *a_s_* and *A_G_* (see below). The gross assimilation was calculated using *C_c_* at the current state of the solver and the equations of Farquhar et al. [12]. The net CO_2_ assimilation *A* was calculated at the end of the simulation as: *A* = *A*_*G*_ − *R*_*d*_. In the last part of the equation *d_a_* is replaced with *d_p_* representing the depth of the photosynthetic tissues (*d*_*p*_ = leaf thickness (m) •[1 − airspaces (%)]).

(7) $$\frac{dL}{dt}=S_{e}\left(1-L\right)-S_{a}A_{G}L$$

The last equation (Eq. (7)) described the negative feedback of sugar accumulation on *a_s_* (Eq. (2)) and *A_G_* during a period of light, with *S_a_* being a factor representing the influence of the rate of accumulation of sugars, and *S_e_* a factor representing the influence of the rate of export of sugars.

Originally in Noe and Giersch [10], this equation only described the negative feedback of sugar export on *A*. The equation was modified to allow recovery of the response of *A_G_* in the absence of light$\left(S_{e}\left(1-L\right)\right)$ and tested using the diurnal data sets described below. The initial simulations showed that the model was able to reproduce the slow decrease of *A* during the day but not the similar decrease observed for *g_s_*. Eq. (2) was therefore modified to include a decrease in *g_s_* during the diurnal period similar to that for *A*.

### 2.2 Solving differential equations

The differential equations were solved using the function lsodes from the R package deSolve (v1.12). The initial absolute tolerance was set to 0.01 for all the outputs and decreased by 10 each time the solver failed to converge. The initial values of the differential equations were chosen based on the observed data. For the pore area, the initial aperture was calculated by solving Eq. (5) for the first recorded *g_s_*.

### 2.3 Parameterization of the model

Parameter values known to impact the diurnal variation of gas exchange were adjusted using the Bayesian inference (see below) to fit the observed data. During the diurnal period, gross CO_2_ assimilation (*A_G_*) was determined by maximum Rubisco activity (*Vc_max_*), maximum electron transport activity (*J_max_*) and the proportion of light absorbed by PSII (α). The day respiration in presence of light (*R_d_*) was used to calculate the net CO_2_ assimilation (*A*). The stomatal conductance to water vapour (*g_s_*) was determined as described above by *a_min_*, *a_max_*, *α_L_* and *k_i_*/*k*_d_. The diurnal variation of *A* and *g_s_* were linked by the negative feedback of sugar export described by *S_a_* and *S_e_*.

Parameter values known to remain unchanged during the diurnal period of measurement or that could be assigned a priori were treated as constants in the model, to facilitate the adjustment, and were chosen from the literature or measured on *Arabidopsis thaliana* (see below). The Michaelis constants and the temperature responses of the Farquhar model were parameterized using values from Walker et al. [13]. The thickness of the leaf was set to 200 μm [14], [15] and the ratio of airspace in the leaf was set to 0.255 [14], [16]. The value of *θ*_*L*_ in equation 2 was chosen at 0.7 by analogy with the similar equation describing the electron transport rate [17]. The boundary layer conductance (*g_b_*) and atmospheric pressure (*P_atm_*) were set to the values provided by the LI-6400XT: 9.29 mol m^−2^ s^−1^ and 101.8 kPa respectively. Estimates of *g_b_* are computed as a function of leaf area and fan speed (LI-6400XT Instruction Manuals, page 3–93). The parameter values describing the membrane properties required to estimate *g_m_* followed that of Von Caemmerer et al. [18]. Stomatal density (*SD*) was assessed on plants grown under the same environmental conditions, on both faces of the leaf (section 1.9). As the model simulated the average response of both faces, the densities of both faces were added and set to 400 mm^−2^. In equation 5, 5.89 μm was used for the depth of the stomatal pore (*l*) [7], the diffusivity of water in air at 25 °C (*D*) was 24.2e^−6^ m^2^ s^−1^, the molar volume of air at 25 °C (*V*) was 0.02446533 m^3^ mol^−1^ [19].

### 2.4 Bayesian inference

The parameter values were estimated using Bayesian inference by comparing the model outputs to recorded gas exchange data [20]. The inference was performed on two objective functions as *A* and *g_s_* were adjusted simultaneously and in this case, there was a risk of bimodal posterior distributions. Monte Carlo Markov Chain (MCMC) using inter-chain adaptation were previously able to sample from bimodal distributions and this technique was thus used to adjust our model (INCA, [21], [22]). To remove any adaptive effect, the final algorithm used to sample the posterior distributions was the random walk metropolis (RWM) with the covariance matrix estimated previously with INCA as an input. The use of Bayesian Inference allowed us to use prior information available in the literature and shown in Table 1. The results of the inference were posterior estimates describing the probability density of the possible parameter values considering the error of prediction and the sensitivity of the model for these parameters and their inter-correlation. The posteriors were represented using quantiles of the posterior distribution at 2.5% and 97.5%, also called credible intervals (similar to a confidence interval). The medians were also shown as the most representative set of parameter values. Two chains with 30,000 iterations were thinned every 2 samples and tested for different criteria: the acceptance rate greater than 15% (to achieve a rapid mixing of the chain), the Effective Sample Size (ESS) > 100 (which is usually enough to describe 95% probability intervals) and the convergence using Monte Carlo Standard Error (MCSE) less than 6.27% (which allows the true mean to be within 5% of the area under a Gaussian distribution around the estimated mean; [23]). The algorithms used to perform the Bayesian inference were implemented using the R software (R Core Team, 2015, v3.2.2).

The accuracy of the model outputs compared to the observed data was assessed using the Root Mean Square Error (RMSE):

(8) $$RMSE=\sqrt{\frac{\sum_{t=1}^{n}{\left(x_{obs,t}-x_{mod,t}\right)}^{2}}{n}}$$

where *x*_*obs*_ and *x*_*mod*_ represents the observed and modelled values to be compared, and *n* the number of observations.

### 2.5 Sensitivity analysis

The sensitivity analysis was performed using the same data set and parameter values estimated previously using Bayesian inference. Each parameter value was altered by ±50%, the impact on the model outputs was summarized by using the daily mean of *A* and *g_s_* divided by the corresponding values obtained using the original parameters values. This resulted in a factor centred on 1 representing the positive or negative impact of the parameter alterations on *A* and *g_s_* (e.g. a factor of 1.1 corresponds to an increase of 10% in the original parameter value).

### 2.6 Plant material

**Fig. 2:**
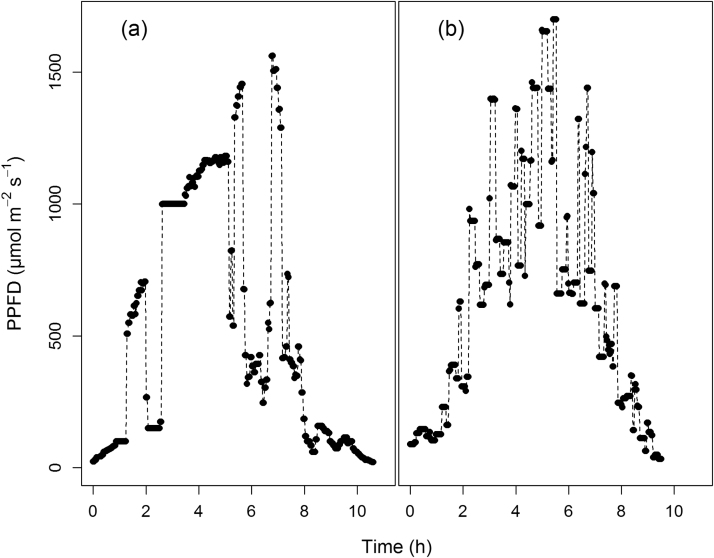
Light regimes a recorded from natural variations at the University of Essex, UK, July 2013, and b simulated using a Gaussian altered with random variations.

*Arabidopsis thaliana* ecotype Col-0 plants were grown in a controlled environment under fluctuating light conditions provided by a Heliospectra LED light source (Heliospectra AB, Göteborg, Sweden). The fluctuating light regime was reconstructed from natural variations in light intensity recorded during a day in July 2013 at the University of Essex, UK (Fig. 2a) assuming a constant spectral distribution. The growth environment was maintained at a relative humidity of 55–65%, a temperature of 21–22 °C and a CO_2_ concentration of 400 μmol mol^−1^. Plants were well watered, and positioning under the Heliospectra light was altered daily. Measurements were taken on the youngest fully expanded leaf.

### 2.7 Model simulations of step change of light

Using the set of parameter values previously estimated, the model was used as a simulator to assess the diversity of the temporal response of *g_s_* after a step change of light. The step change was performed by initially setting the light to an intensity of 100 μmol m^−2^ s^−1^ for 30 min followed by a step increase in intensity to 1000 μmol m^−2^ s^−1^ for 2 h. All other environmental conditions were kept constant, with the [CO_2_] maintained at 400 μmol mol^−1^ and a leaf temperature of 25 °C. Parameter values of *SD*, *k_i_* and α*_L_* were increased or decreased by a factor of 2 to illustrate their impact on temporal responses of *g_s_*, *A* and *W_i_*. The rate of change of *g_s_* was estimated using a linear regression on the first 10 min of the variation. The impact of the temporal response of *g_s_* on *A* and *W_i_* was characterised by calculating the percentage of variation of the mean *A* and *W_i_* during the 2 h after the step change of light.

### 2.8 Leaf gas exchange

Photosynthesis gas exchange parameters (*A* and *g_s_*) were measured using a Li-Cor 6400XT portable gas exchange system (Li-Cor, Lincoln, Nebraska, USA), with dew point and vapour pressure deficit maintained *via* a Li-Cor 610 portable dew point generator. Throughout the measurement cuvette, conditions were maintained at a CO_2_ concentration of 400 ppm (corresponding to ambient growth), leaf temperature of 25 °C, and a leaf to air water vapour pressure deficit of 1 (±0.2) kPa. The largest fully expanded mature leaf was used, with *A* and *g_s_* allowed to stabilize under the controlled cuvette conditions for a minimum of 30 min. At this point, the automatic 12 h light program (mirroring that of the growth conditions, Fig. 2a) was started, with *A* and *g_s_* recorded every 2 min. *W_i_* was calculated as *A*/*g_s_*. For the model validation, the same measurement conditions were used in the cuvette with the exception of the light that reproduced a different pattern of light intensity (Fig. 2b).

### 2.9 Stomatal density

Following the dental impression methods of Weyers & Johansen (1985), negative impressions of the ab- and ad-axial leaf surface were made using Xantoprene Polysiloxane precision material (Heraesus Kulzer Ltd). Positive impressions were produced by coating nail varnish on the dry polymer and used to count the number of stomata in 9 field of view using an Olympus BX60 light microscope (Olympus Europa, Southend-On-Sea, UK). The area of view was measured using an eyepiece graticule to express the number of stomata by mm^−2^. Stomatal impressions of the leaf surface were taken on the same leaves as gas exchange measurements were conducted.

## 3 Results

### 3.1 Bayesian inference

The two chains (describing the posterior distributions) resulting from the Bayesian inference successfully converged and were used to calculate the credible intervals of each parameter shown in Table 1. The credible interval of *a_min_* was significantly higher and not overlapping the prior distribution, which was not the case for *a_max_*, suggesting that the observed data were informative for *a_min_* but not for *a_max_*. The model estimated α*_L_* precisely, despite the fact that the prior distribution covered a large range of possible values, suggesting that the model was particularly sensitive to this parameter. The time constants, *k_i_* and *k*_*d*_, displayed a strong asymmetry with values almost 3 times higher for *k_i_* compared to *k*_*d*_. *Vc_max_* and *J_max_* values were sensibly higher than the prior means estimated from the literature, however the prior distribution and the credible interval overlapped, suggesting that these values were still in the range observed in the literature.

**Fig. 3:**
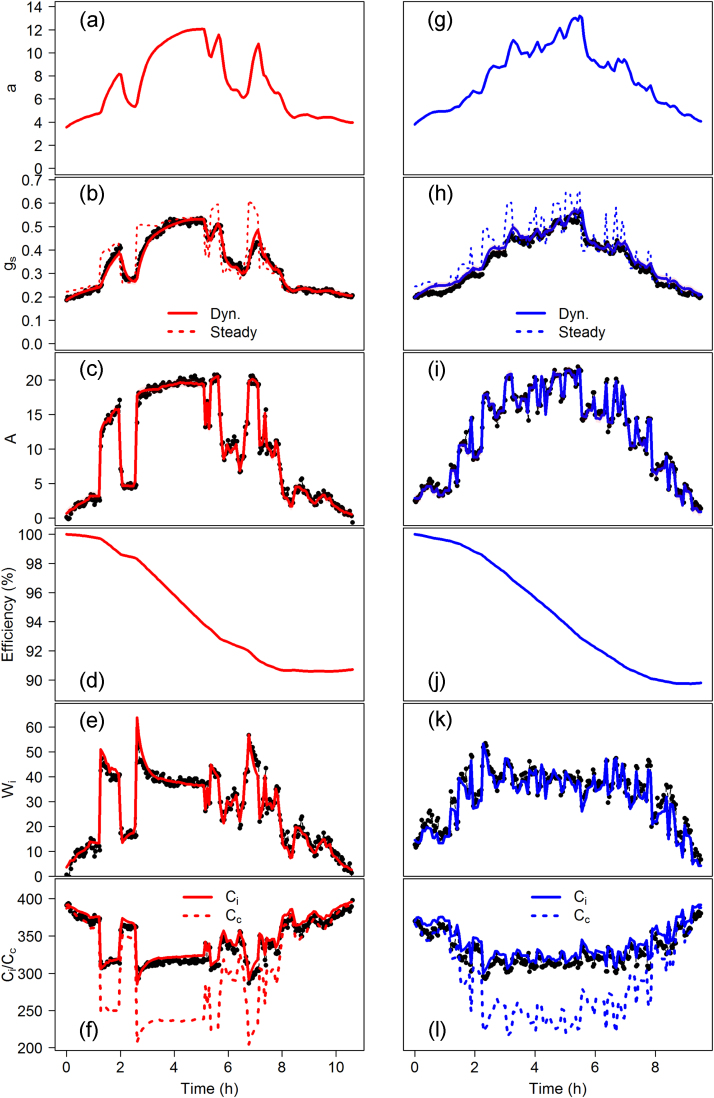
Modelled (solid & dashed lines) and observed (black circles) diurnal variations in gas exchange under two different light regimes (Fig. 2). a–f Modelled data (red lines) were fitted to observations (black circles) using Bayesian inference to estimate the most credible set of parameter values under the light regime described in Fig. 2a. g-l Modelled data (blue lines) were simulated using the same set of parameter values and the light regime describe in Fig. 2b. The red and blue dashed line in b and h represented the steady state *g_s_* target (Steady) if conditions remained constant, whilst solid lines represent the dynamic outputs. (For interpretation of the references to colour in this figure legend, the reader is referred to the web version of this article.)

The medians of the posterior estimates shown in Table 1 were used to represent the most representative prediction of the model (Fig. 3a–f). Under a diurnal fluctuating light regime, our dynamic model accurately described *g_s_* (Fig. 3b, RMSE: 0.014 mol m^−2^ s^−1^; Suppl. Fig. S1a, R^2^:0.984) and *A* (Fig. 3c, RMSE: 0.6 μmol m^−2^ s^−1^; Suppl. Fig. S1b, R^2^:0.993). Consequently, the rapid variations of *W_i_* were also accurately reproduced (Fig. 3e, RMSE: 2.58 μmol mol^−1^; Suppl. Fig. S1c, R^2^:0.97). By adjusting the model to the observed *g_s_*, our ‘big stoma’ approach allowed us to predict the average behaviour of the stomata with a variation of the pore area from 4 to 14 μm^2^ (Fig. 3a). Under these environmental conditions *A* and *g_s_* displayed a decrease in efficiency of approximately 8% at the end of the diurnal period (Fig. 3d), which was described by *S_e_* and *S_a_*. The model seemed to be less sensitive to *S_e_* as the credible interval was much larger than the one of *S_a_*. The model successfully describes the variation of *C_i_* but also predicts the variations of *C_c_* that showed a larger variation compared to *C_i_* due to low *g_m_* (Fig. 3f).

Fig. S1 Modelled (Mod.) vs observed (Obs.) stomatal conductance to water vapour (*g_s_*, mol m^−2^ s^−1^), net CO_2_ assimilation (A, μmol m^−2^ s^−1^) and intrinsic water use efficiency (*W_i_*, μmol(CO_2_)/mol(H_2_O)) measured under: a–c light regime described in Fig. 2a for the model parameterization, d–f light regime describe in Fig. 2b for the model validation. Solid lines and dashed lines represented the 1:1 lines and linear regressions respectively. The coefficient of determination (R^2^) was derived from the linear regression.

### 3.2 Model validation

To validate the model, the same plant and set of parameter values (Table 1) were used to predict *A*, *g_s_* and *W_i_* under a different light regime (Fig. 2b). Our dynamic model was able to accurately predict the observed gas exchanges (Fig. 3g-l): *g_s_* (Fig. 3h, RMSE: 0.023 mol m^−2^ s^−1^; Suppl. Fig. S1d, R^2^:0.983), *A* (Fig. 3i, RMSE: 0.69 μmol m^−2^ s^−1^; Suppl. Fig. S1e, R^2^:0.988) and *W_i_* (Fig. 3k, RMSE: 2.95 μmol mol^−1^; Suppl. Fig. S1f, R^2^:0.961). Compared to the rapidity of the stomatal response (*k_i_*: 24 min and k_*d*_: 9 min), the light variations were too fast (on average a change every 8 min) to observe the complete stomatal response and, as a consequence, *a* followed the general tendency of the light intensity (Fig. 3g). The efficiency of *A* and *g_s_* during the diurnal period showed a decrease of around 10% similar to that of the estimate during the other diurnal period (Fig. 3j).

### 3.3 Sensitivity analysis

**Fig. 4:**
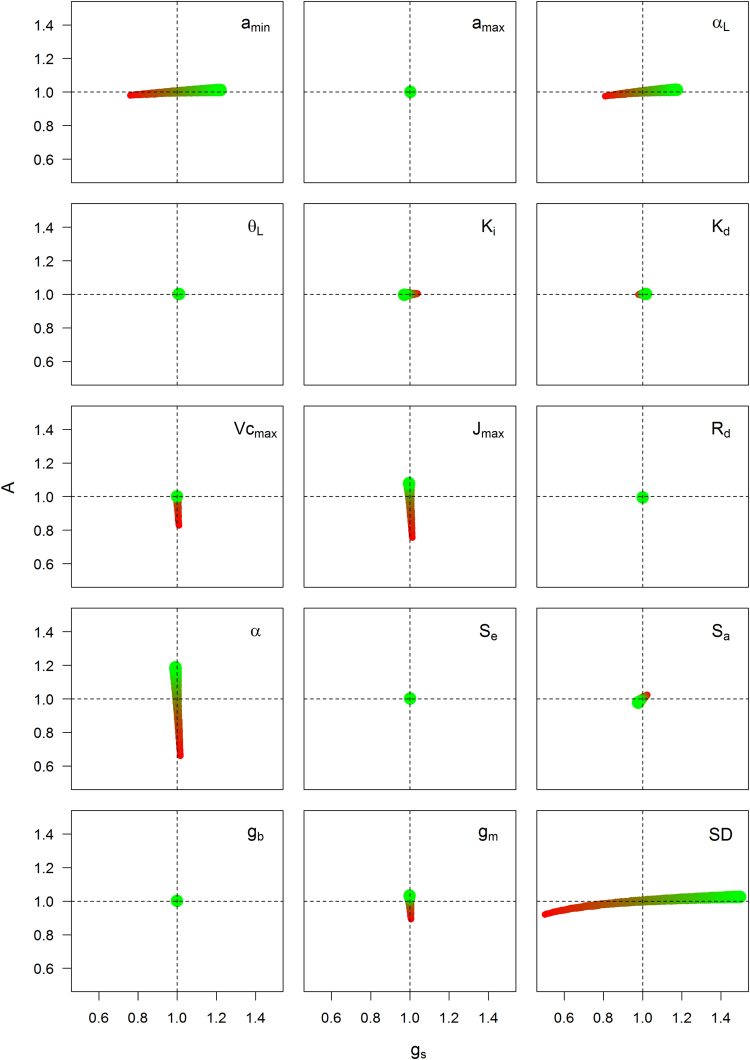
Relative variation of the daily mean net assimilation (*A*) and daily mean stomatal conductance to water vapour (*g_s_*) using parameter values ranging ±50% around the estimated parameters from Table. 1. The size and the colour of the circles are proportional to the deviation of the parameter values (small and red circles correspond to decreased parameter values whilst large green circles correspond to increased parameter values). The length and direction are relative to the impact of the parameter on *A* and *g_s_*.The simulated daily mean of *A* and *g_s_* for each parameter set was divided by the mean values of *A* and *g_s_* determined using the parameters in Table 1. The (1,1) coordinate showed similar daily mean values of *A* and *g_s_* compared to values simulated using the parameter set described in Table 1. (For interpretation of the references to colour in this figure legend, the reader is referred to the web version of this article.)

A sensitivity analysis of the model was undertaken to illustrate the impact of varying individual parameters on *A* and *g_s_* (Fig. 4). The parameters that displayed the most sensitivity for *g_s_* were (ordered by importance): *SD*, *a_min_*, α_L_, *k_i_*, and *S_a_*. For *A*, the parameters were (ordered by importance): α, *J_max_*, *Vc_max_*, *g_m_*, *SD, a_min_*, α_L_ and *S_a_*, showing the interdependence of *A* and *g_s_*. Even if the model was sensitive to the variation of a parameter, this variation would not necessarily improve *W_i_*. For example, *Vc_max_* only displayed variation in *A* that resulted in a decrease in *W_i_* suggesting that under the conditions used here *A* is not limited by CO_2_ diffusion. The greatest impact on both *A* and *g_s_* was observed when *SD* was decreased, while increasing photosynthetic capacity (*Vc_max_* and *J_max_*) by 50% only improved *A* by 10% under the environmental conditions used here.

### 3.4 Impact of stomatal characters on *g_s_*, *A* and *W_i_* temporal responses

**Fig. 5:**
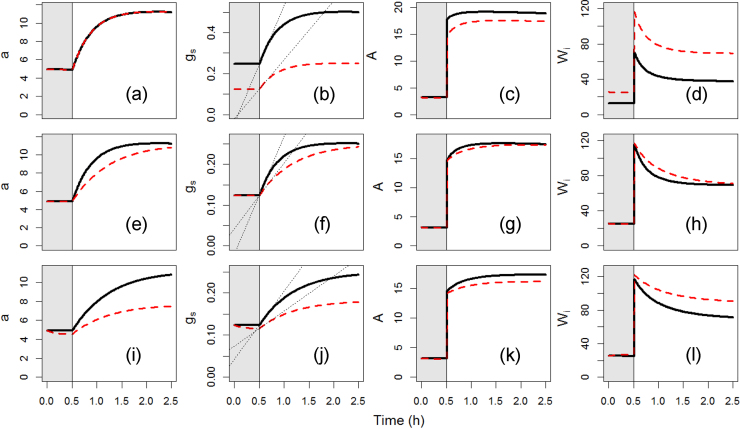
Simulation of the temporal response of *a*, *g_s_*, *A* and *W_i_* with different alterations on *SD*, *k_i_*, and α*_L_* under a step change of light from 100 (shaded area) to 1000 (white area) μmol m^−2^ s^−1^. a-d Impact of a variation of the stomatal density (*SD*) from 400 (black line) to 200 mm^−2^ (red dashed line). e-h Impact of a variation of the time constant for stomatal aperture (*k_i_*) from 1430 (black line) to 2860s (red line). i-l Impact of a variation of the slope of the relationship between the pore area and the light (α*_L_*) from 0.77 (black line) to 0.38 μm^2^/μmolm^−2^s^−1^(red line). The dotted lines on (b, f, j) represent the rates of *g_s_* variation for the first 10 min of the step change estimated by linear regression. (For interpretation of the references to colour in this figure legend, the reader is referred to the web version of this article.)

In order to determine the impact of stomatal characters on the temporal response of *g_s_*, *A* and *W_i_*, the influence of the rapidity of the stomatal response (*k_i_*) and the steady states *g_s_* (SD, *α_L_*) to a step increase in light intensity were examined (Fig. 5). The initial slope of the *g_s_* response displayed large differences (e.g. 0.58–0.29 mol m^−2^ s^−2^, Fig. 5b) even if the rapidity of increasing stomatal aperture did not change (Fig. 5a). The different temporal responses of *g_s_* driven by *SD*, *k_i_* and α*_L_* limited *A* by 8.8% (Fig. 5c), 2% (Fig. 5g) and 6.1% (Fig. 5k) respectively. The impact on *W_i_* was due to a larger decrease in *g_s_* rather than *A* following the step increase in light intensity, with an increase of *W_i_* by 82% (Fig. 5d), 7.4% (Fig. 5h) and 19.2% (Fig. 5l) respectively.

## 4 Discussion

Over different diurnal light regimes, our new dynamic model was able to incorporate the rapid fluctuations in light and accurately predicted *g_s_*, *A* and *W_i_*. To our knowledge, this is the first attempt to model stomatal behaviour and scale it up to leaf level to predict gas exchange under dynamic light regimes. This ‘big stoma’ approach to model g_s_ provides a direct estimation of the rapidity of the stomatal response, whereas most current models consider only an instantaneous response [3]. Moreover, the simulated average pore area (*a*) predicted by the model was in a similar range to those previously reported in the literature [24], [25]. Previous studies have attempted to directly model temporal responses of *g_s_* [4], [5], but our approach models the speed of increasing pore area rather than the speed of g_s_ increase, and is therefore more mechanistic and biologically relevant when describing the dynamics of gas exchange. Although we have validated the model using responses to fluctuations in light intensity, other environmental variables (e.g relative humidity) could be included in the future and would greatly improve the predictive power of the model.

Our model also incorporates negative feedback on *A* and *g_s_* that revealed a 10% decrease in both by the end of the diurnal period. Noe and Giersch [10] suggested that the accumulation of sugars from photosynthesis could negatively regulate *A*. The decrease in g_s_ and therefore subsequent decrease in guard cell turgor, could be related to the decrease of *A* [26], [27], [28]. Such mechanisms for negative feedback are not well documented but are important for breeders, as any increase in photosynthetic capacity could lead to a great decrease in efficiency over the diurnal period. By accurately predicting diurnal gas exchange and dissecting *W_i_* into the key parameters controlling dynamic responses, we believe our model will provide an important tool for future breeding strategies to identify targets for improved *W_i_* under different environmental conditions.

Previous studies have shown spatial heterogeneity in stomatal distribution and behaviours [29], [30], [31], [32] that have not been included in the model. Instead, the model predicted the behaviour of a stoma that is representative of the sum of individual stomatal responses over the entire leaf surface. This simplification did not appear to impact on the quality of the predictions and it is difficult to assess the temporal and spatial heterogeneity in stomatal response; therefore, further investigations would be required to determine if and how such variations would impact model outputs.

Stomatal density (SD) was shown to be the most sensitive stomatal parameter for improving *W_i_*, with decreases in SD resulting in a substantial reduction in g_s_ but minimal impact on *A*. This is in agreement with the work of Franks et al. [33], who showed significant variation of *W_i_* in Arabidopsis mutants with different SD. The Bayesian inference and the sensitivity analysis revealed that the model was not sensitive to changes in *a_max_*, as this value was not reached at any point over the diurnal period. In contrast, *a_min_* and *α_L_* were important determinants of *W_i_* but are rarely reported or examined in this context in the literature. The model predicts that, under dark conditions, *a_min_* is statistically greater than zero resulting in significant nocturnal transpiration, which has been reported previously [34] and has implications for overall plant water use. As SD cannot change over the diurnal period, *α_L_* could be an important determinant of plant water use by adjusting the magnitudes of change in *g_s_* as a function of the environment.

Although the coefficient of light absorption by PSII (α) was an important determinant of *A* and *W_i_* in the field, this parameter does not show a large diversity; therefore, *Vc_max_*, *J_max_* and *g_m_* are the most promising targets for improving *A*. The model predicts that increasing *g_m_* could lead to similar improvements in *A* and *W_i_* [35] as those observed when *Vc_max_* and J_max_ were increased through manipulating enzymes associated with the Calvin cycle (e.g. [36]).

The sensitivity analysis performed here should be seen as a tool to determine the key parameter(s) for improving *W_i_* under specific environmental conditions (e.g. fluctuating light intensity). Indeed, the parameter values estimated with the Bayesian inference are specific for this study and the variation around the determined parameter may change between individuals and/or species. For example, an individual with different photosynthetic capacity (*Vc_max_*, *J_max_*) or SD will exhibit different thresholds of A limitations (Fig. 5c) and the results of the sensitivity analysis may also vary. To generalize the sensitivity analysis, ranges of biologically possible parameter values and the interactions between them (e.g. relationship between size of the pore and SD) should be used; however, to our knowledge, this information is not yet fully available in the literature.

Our model simulations clearly showed that the temporal response of g_s_ is the product of several parameters (SD, *α_L_* and *k_i_*/*k*_d_), and the rapidity of the stomatal response cannot be interpreted by comparing the initial slope of variation in *g_s_*, as is often reported in the literature [37], [38]. Changes in the initial slope of the *g_s_* response are correlated with SD, *α_L_* and *k_i_*/*k*_d_ and therefore are not only indicative of the speed of changes in stomatal aperture. In terms of gas diffusion, stomatal size constrains the maximum pore area but the observed pore area also depends on the sensitivity of the guard cell (*α_L_*) responses to light intensity. As a result, it is theoretically possible to have large stomata that even at high light retain a small pore area. Considering the relationship between stomatal size and density [39], our results suggest that having a high density of small stomata will result in fast changes in g_s_ but also in a high average g_s_ value through the diurnal period, decreasing *W_i_*.

The model simulations also revealed large variations in *W_i_* (>80%) when the parameter values controlling the temporal response of *g_s_* were altered. Indeed, the decrease in *A* due to greater limitation of CO_2_ diffusion was minimal compared to the improvement in *W_i_*. The importance of the temporal response of *g_s_* was highlighted previously [40], [41], but not the importance of the additive effects of parameter interactions on *A* and *W_i_* as revealed by our model. Therefore, our model could be used to find the optimal parameter set that would decrease *g_s_* without impacting *A* and maximizing *W_i_* to direct future breeding and research programmes to identify plants with reduced water usage, but the same or greater productivity.

## 5 Conclusion

We assessed and validated a new dynamic model of leaf gas exchange that accurately predicts *g_s_*, A, and *W_i_* under fluctuating light regimes such as those experienced by plants in the field. The model uses a unique framework that takes into account leaf anatomy, biochemistry and the physiological responses of stomata on *W_i_*. The model revealed important negative feedback controls on *A* and *g_s_* towards the end of the diurnal period that resulted in decreases in *A* with implications for overall plant productivity. Importantly, the model enabled *W_i_* to be dissected into key parameters such as stomatal sensitivity to light (*α_L_*) and minimal pore area under dark conditions (*a_min_*) that have previous been neglected and could provide new and realistic targets for future improvements in crop water use efficiency.

## Figures and Tables

**Table 1:** Parameters of the dynamic gas exchange model adjusted with Bayesian inference: mean and standard deviation (SD) of the prior values estimated from the literature (see footnotes) and quantiles at 2.5%, 50% and 97.5% of the credible interval of each posterior distribution estimated using Bayesian inference.

	unit	Prior		Posterior		
		Mean	SD	2.5%	50%	97.5%
*a_min_^1^*	μm^2^	3.56	0.2	4.03	4.16	4.27
*a_max_^2^*	μm^2^	100	10	72.15	93.24	113.87
*_αL_*	$\frac{\mu {\text{m}}^{2}}{\mu \text{mol}{\text{m}}^{-2}{\text{s}}^{-1}}$	0	10	0.742	0.767	0.793
*k_i_*	s	100	1e4	1290	1430	1580
*k*_*d*_	s	100	1e4	463	541	627
*Vc_max_^3^*	μmolm^−2^s^−1^	65.5	22.6	89.8	99.3	128.8
*J_max_^3^*	μmolm^−2^s^−1^	102.4	21.9	180.5	186.4	193
*R_d_*	μmolm^−2^s^−1^	0	10	0.01	0.14	0.37
α	$\frac{\mu \text{mol}\left(electron\right)}{\mu \text{mol}\left(photon\right)}$	0	1	0.177	0.183	0.192
*S_e_*		0	10	0.263e^−6^	8.486e^−6^	32.904e^−6^
*S_a_*		0	10	0.207e^−6^	0.280e^−6^	0.385e^−6^

1. Parameter estimated using the observed stomatal conductance under dark conditions.

2. Parameter estimated from Dow et al. [7]

3. Parameter estimated by averaging published values for Col-0 by Flexas et al. [42], Tholen et al. [14], Heckwolf et al. [43], Cousins et al. [44], Sade et al. [45].
