# Supplementary material for: Modelling water use efficiency in a dynamic environment: An example using Arabidopsis thaliana
Source: Plant Sci. 2016 Oct;251:65–74. doi: 10.1016/j.plantsci.2016.06.016 (PMC5038844; doi:10.1016/j.plantsci.2016.06.016)
Supplement: Fig. S1 — Modelled (Mod.) vs observed (Obs.) stomatal conductance to water vapour (gs, mol m−2 s−1), net CO2 assimilation (A, μmol m−2 s−1) and intrinsic water use efficiency (Wi, μmol(CO2)/mol(H2O)) measured under: a–c light regime described in Fig. 2a for the model parameterization, d–f light regime describe in Fig. 2b for the model validation. Solid lines and dashed lines represented the 1:1 lines and linear regressions respectively. The coefficient of determination (R2) was derived from the linear regression. [file mmc1.docx]

**Supplementary data**


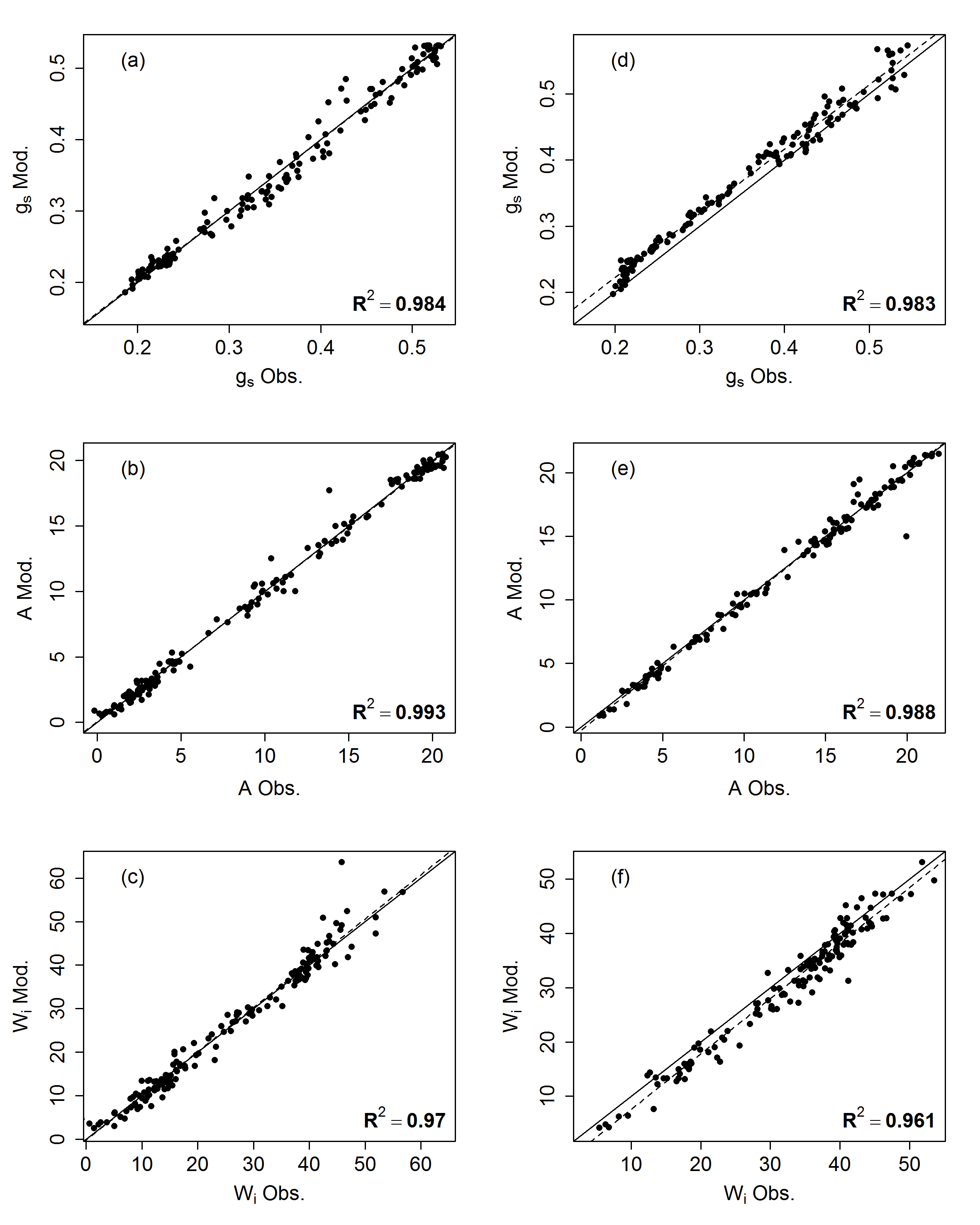


Supplemental Figure 1: Modelled (Mod.) *vs* observed (Obs.) stomatal conductance to water vapour (*g_s_*, mol m^-2^ s^-1^), net CO_2_ assimilation (*A*, µmol m^-2^ s^-1^) and intrinsic water use efficiency (*W_i_*, µmol(CO_2_)/mol(H_2_O)) measured under: **a-c** light regime described in Fig. 2a for the model parameterization, **d-f** light regime describe in Fig. 2b for the model validation. Solid lines and dashed lines represented the 1:1 lines and linear regressions respectively. The coefficient of determination (R^2^) was derived from the linear regression.
